# A case of hemodiafiltration dialysis preventing progression of acute kidney injury in a patient with hypermyoglobinemia in one kidney: a case report

**DOI:** 10.1186/s13256-025-05138-w

**Published:** 2025-03-20

**Authors:** Toru Minamiyama, Shozo Yoshida, Yosuke Mizuno, Hiroki Kitagaki, Kaori Kimura, Yoshihito Naito, Akihiro Uchida, Ayumi Kuroda, Yuki Kawasaki, Ayane Nishio, Hirotsugu Fukuda, Genki Yoshimura, Ryo Kamidani, Takahito Miyake, Norihide Kanda, Hideshi Okada

**Affiliations:** 1 Advanced Critical Care Center, Gifu University Hospital, Gifu, Japan; 2 Abuse Prevention Center, Gifu University Graduate School of Medicine, Gifu, Japan; 3 Center for One Medicine Innovative Translational Research, Gifu University Institute for Advanced Study, Gifu, Japan; 4 Department of Emergency and Disaster Medicine, Gifu University Graduate School of Medicine, 1-1 Yanagido, Gifu, 501-1194 Japan

**Keywords:** Hypermyoglobinemia, Rhabdomyolysis, Acute renal failure, Emergency hemodiafiltration, Intermittent renal replacement therapy

## Abstract

**Background:**

Rhabdomyolysis is a clinical syndrome resulting from skeletal muscle damage and the release of its breakdown products into the bloodstream. It can range from asymptomatic cases to severe conditions such as acute kidney injury. Although the release of myoglobin (molecular weight 17.2 kDa) into the blood is associated with the progression to acute kidney injury, there is no established method to prevent it. Here, we report a case of hypermyoglobinemia due to rhabdomyolysis caused by reperfusion injury following acute limb ischemia, where early renal replacement therapy was deemed effective.

**Case presentation:**

The patient, a 73-year-old Japanese male, had a history of right nephrectomy due to trauma. At 2 years prior, he underwent bypass surgery connecting the subclavian artery to the bilateral femoral arteries to treat lower limb arteriosclerotic occlusive disease. In this case, he presented to another hospital with sudden right lower limb pain and was referred to our hospital with a diagnosis of acute occlusion of the right lower extremity artery. After emergency endovascular thrombectomy, hemodiafiltration was initiated on the second day due to rhabdomyolysis and hypermyoglobinemia. The patient developed compartment syndrome in the affected limb and underwent an emergency fasciotomy. Despite a further increase in myoglobin levels, his urine output remained stable, and creatinine levels stayed within the normal range. On the 6th day of admission, he was successfully weaned off hemodiafiltration. Following negative pressure wound treatment for compartment syndrome, a skin graft was performed, and the wound was closed. The patient was transferred for rehabilitation on the 35th day.

**Conclusion:**

This case illustrates that early initiation of blood purification therapy can prevent the progression of acute kidney injury triggered by hypermyoglobinemia in rhabdomyolysis. Early intervention with intermittent hemodiafiltration may effectively prevent renal failure in such cases.

## Background

Rhabdomyolysis is a clinical syndrome caused by skeletal muscle damage and the release of its breakdown products into the circulation, ranging from asymptomatic to severe cases, such as acute kidney injury (AKI) [1]. The release of myoglobin (Mb; molecular weight: 17.2 kDa) into the blood is involved in the development of AKI. Although renal replacement therapy (RRT) is not the first choice for AKI prevention, the removal of myoglobin and other inflammatory substances and early intervention may reduce its risk [2–5]. In the present study, we report a case of hypermyoglobinemia owing to rhabdomyolysis caused by reperfusion injury after acute limb ischemia that responded to early RRT.

## Case presentation

The patient was a 73-year-old Japanese man who previously underwent surgery for primary lung cancer and was receiving anticancer drugs. He had a history of right nephrectomy following trauma. Additionally, two years prior, he had bypass surgery connecting the subclavian artery to the bilateral femoral arteries to treat lower limb arteriosclerotic occlusive disease. In this case, he initially presented at another hospital with sudden right lower limb pain and was subsequently referred to our hospital, where he was diagnosed with acute occlusion of the right lower extremity artery. Upon arrival at our hospital, his Glasgow Coma Score (GCS) was 15 points (GCS E4V5M6). The patient’s vital signs were as follows: blood pressure, 194/73 mmHg; heart rate, 107 beats/minute; respiratory rate, 21 breaths/minute; SpO_2_, 98% (room air); and temperature, 36.2℃. In the right lower extremity, blood pressure and SpO_2_ were unmeasurable, and the skin was pale with spontaneous pain. Arterial blood gas and laboratory test results are shown in Table 1.

**Table 1:** Laboratory findings at the time of admission

Biochemistry			Complete blood count		
Total protein	6.9	g/dL	White blood cells	12,470	/µL
Albumin	4.5	g/dL	Red blood cells	3.99 × 10^6^	/µL
Creatinine kinase	125	IU/L	Hemoglobin	14.1	g/dL
AST	35	IU/L	Hematocrit	40.7	%
ALT	13	IU/L	Platelet	178 × 10^3^	/µL
LDH	254	IU/L			
γ-GTP	38	IU/L	Coagulation status		
Amylase	90	IU/L	APTT	20.7	sec
Total bilirubin	0.8	mg/dL	PT-INR	0.79	
Direct bilirubin	0.2	mg/dL	Fibrinogen	241	mg/dL
Creatinine	0.70	mg/dL	FDP	131.9	µg/mL
BUN	10.9	mg/dL	D-dimer	29.4	µg/mL
Sodium	136	mEq/L			
Potassium	3.7	mEq/L	Arterial Blood Gas		
Chloride	100	mEq/L	F_I_O_2_	0.40	
Magnesium	1.7	mg/dL	pH	7.348	
Calcium	9.3	mg/dL	P_a_CO_2_	42.4	mmHg
Glucose	202	mg/dL	P_a_O_2_	199.0	mmHg
HbA1c	5.5	%	HCO_3_^−^	23.3	mmol/L
CRP	0.03	mg/dL	Base excess	−2.3	
			Lactate	4.9	mmol/L
			Anion gap	12.6	

AST, aspartate aminotransferase; ALT, alanine aminotransferase; LDH, lactic acid dehydrogenase; γ-GTP, γ-glutamyl transpeptidase; BUN, blood urea nitrogen; HbA1c, hemoglobin A1c; CRP, C-reactive protein; APTT, activated partial thromboplastin time; PT-INR, prothrombin time-international normalized ratio; FDP, fibrin degradation product; F_I_O_2_, fraction of inspiratory oxygen

**Fig. 1:**
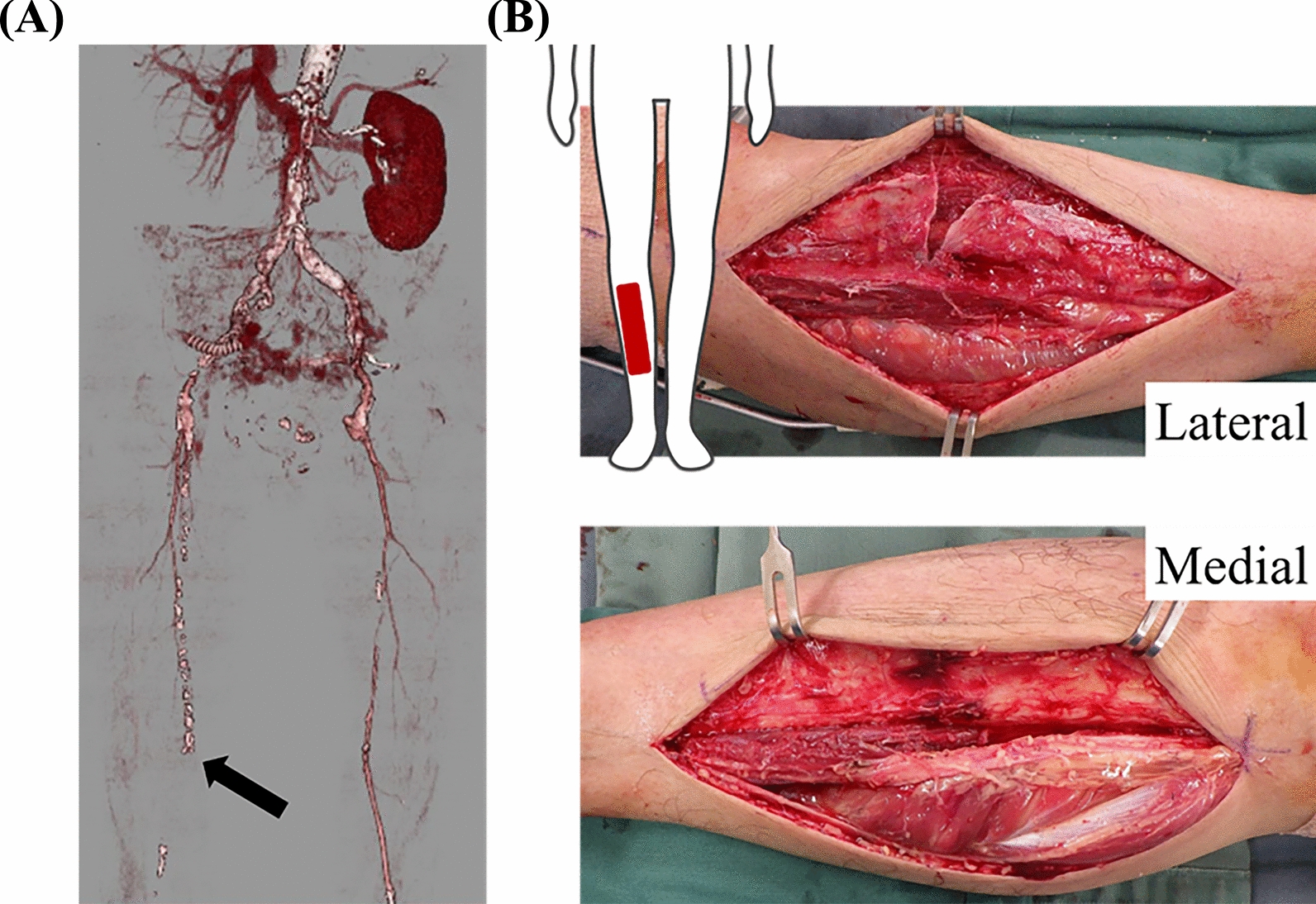
Diagnostic image findings. **a** Contrast-enhanced computed tomography scan at the time of transport to the hospital. Occlusion beyond the right femoral artery was observed (closed arrow). **b** On the 2nd day, the patient developed compartment syndrome in the right lower leg and underwent an emergency fasciotomy. No obvious necrotic tissue was found

**Fig. 2:**
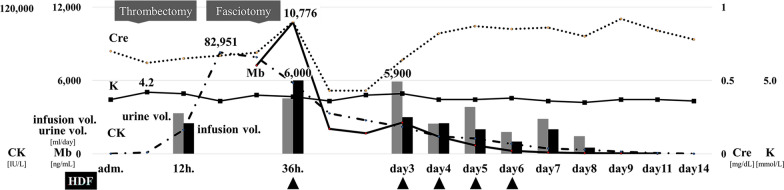
Post-hospitalization course. After transport, emergency thrombectomy was performed, and hemodiafiltration was started on the second day due to high myoglobin and creatine kinase levels. On the same day, the patient developed compartment syndrome and underwent emergency fasciotomy. Despite a further increase in myoglobin and creatine kinase levels, urine output remained stable, and creatinine and potassium levels stayed within the normal range. The patient was weaned off hemodiafiltration on the 6th day. Circulation was stable during the disease. Gray bars indicate urine volume (mL/day) and black bars indicate infusion volume (mL/day). Dialysis prescription: duration of session, 8 hours; mode, postdilution-HDF; Q_D_, 500 mL/minute; Q_B_, 200 mL/minute; Q_S_, 3.6L/h; filtration membrane, Nikkiso FDZ-21 surface area 2.1 m^2^

Contrast-enhanced computed tomography revealed an occlusion beyond the right femoral artery (Fig. 1, left). The patient’s clinical course after admission is shown in Fig. 2. Following emergency endovascular thrombectomy on the first day, hemodiafiltration (HDF) was initiated on the 2nd day due to the development of rhabdomyolysis and hypermyoglobinemia. Blood tests on this day revealed high levels of creatine kinase (CK, 82,951 IU/L) and myoglobin (7,237 ng/mL). A polyester polymer alloy (PEPA) dialyzer was used for HDF. Additionally, the patient developed compartment syndrome due to elevated internal pressure in the lower leg and underwent emergency fasciotomy (Fig. 1, right). Although the myoglobin level temporarily increased to 10,776 ng/mL on the 6th day of admission, the patient was successfully weaned off HDF. Throughout the treatment, the patient’s circulation remained stable, his urine output was maintained, and creatinine levels stayed within the normal range. After receiving negative pressure wound treatment for compartment syndrome, a skin graft was performed, and the wound was closed. The patient was transferred for rehabilitation on the 35th day.

## Discussion and conclusion

Acute limb ischemia occurs due to a sudden decrease in arterial perfusion in the extremities, necessitating rapid revascularization such as endovascular treatment or surgery. While the occurrence of acute limb ischemia is rare, its incidence is higher in patients with preexisting advanced peripheral arterial disease, as seen in this case. Nontraumatic cases, such as the present case, can be broadly classified as embolic or thrombotic; however, the most common etiology is acute thrombosis of bypass grafts or stents [6]. Ischemia-damaged skeletal muscle cells are placed in an anaerobic environment where aerobic metabolism and oxidative phosphorylation are markedly reduced, eventually leading to cell death. Reperfusion releases electrolytes, CK, and Mb [7]. Rhabdomyolysis is a clinical syndrome caused by skeletal muscle damage and the release of these substances into the circulation. It can range from asymptomatic to severe, including compartment syndrome and AKI [1, 8]. Rhabdomyolysis diagnosis is usually made by confirmation of elevated blood levels of components present in muscle cells, such as CK and Mb (> five times the upper limit of normal for CK) [2, 9, 10]. However, CK levels are highly variable, and their effects on individuals differ widely, while the nephrotoxicity caused by CK remains unclear. In contrast, some studies suggest that Mb is not a reliable diagnostic indicator [2]; however, nephrotoxicity has been indicated for some time [11] and is considered the main cause of AKI triggered by rhabdomyolysis [12]. This suggests its usefulness as a biomarker [13], and since there have been reports that AKI incidence was significantly higher in cases with peak serum Mb levels > 15,000 μg/L, it is considered a reliable indicator for determining disease status [1, 10].

Renal replacement therapy (RRT) is not the first choice for preventing AKI progression due to rhabdomyolysis, particularly in patients with preserved urine output [1]. However, when urine output is reduced, Mb tends to form a column with uromodulin in the thick ascending leg, inhibiting renal excretion of Mb and promoting tubular damage [1], indicating that the removal of Mb and other inflammatory substances and early intervention may reduce this risk [2–5]. Although reports indicate that continuous RRT is more stable than intermittent RRT in terms of circulatory dynamics [3], many patients with rhabdomyolysis maintain stable circulatory function after adequate infusion load, making intermittent RRT a viable option. However, it remains unclear whether hemodialysis or HDF is more effective.

Although we administered adequate infusions for rhabdomyolysis from the outset [14], Mb levels continued to rise. Despite maintaining urine output, we determined that early intervention beyond infusions was necessary. Bicarbonate and mannitol have been demonstrated to be ineffective in treating rhabdomyolysis [14], and loop diuretics may exacerbate Mb precipitation and worsen distal tubular obstruction [15]. While some guidelines suggest initiating RRT for rhabdomyolysis on the basis of conventional indications for AKI and the degree of renal dysfunction [16], other studies indicate that early HDF may reduce the risk of AKI [5]. The patient had previously undergone bypass grafting for lower extremity arteriosclerosis obliterans and was prone to thrombosis, with compartment syndrome postoperatively. Additionally, the patient had undergone nephrectomy owing to a previous traumatic injury, resulting in a relatively low renal reserve. This condition increased the likelihood of declining urine output, making the patient more susceptible to AKI and its progression to chronic renal failure. Therefore, we decided to initiate RRT. We selected HDF (dialysis prescription shown in Fig. 2) because it is the standard method employed in our intensive care unit. Given the molecular weight of Mb, we expected a higher removal rate. In our experience, patients with Mb levels > 3,000 ng/mL or with a tendency to develop AKI often started HDF on the 2nd day of treatment. We decided to withdraw HDF based on the patient’s vital signs; urine volume; and Cre, CK, and Mb values as well as their trends. In our department, Mb values are used as an index to determine the effectiveness of treatment, and trends in CK values are used only as a reference. Although CK and Mb values can be measured at any time in our hospital, the effects of CK on the body, such as nephrotoxicity, as described above, remain unclear, and we have found no consensus on using CK values as an indicator for induction and withdrawal of blood purification [17, 18]. Regarding RRT, because the patient’s vital signs were stable and urine output was maintained from the beginning, we judged that the patient’s hemodynamics were stable and decided to perform intermittent HDF after consultation with a clinical engineer. For efficient Mb removal, a PEPA dialyzer (Nikkiso, FDZ-21, surface area of 2.1 m^2^) was selected as the filtration membrane. Throughout the treatment, urine output remained sufficient, and Cre levels stayed within the normal range. As demonstrated in this case, early initiation of HDF may effectively prevent AKI progression in patients with hypermyoglobinemia and reduced renal reserve. Additionally, intermittent RRT can be sufficient when the patient’s hemodynamic condition is stable.

In conclusion, we encountered a case of acute blood purification therapy for hypermyoglobinemia caused by rhabdomyolysis following reperfusion injury after acute limb ischemia. Early intervention with hemodialysis may help prevent AKI progression in such cases. Furthermore, this study suggests that intermittent hemodiafiltration dialysis can be an effective treatment option.

## Data Availability

The datasets obtained and analyzed in the current study are available from the corresponding author upon reasonable request.

## Abbreviations

AKI: Acute kidney injury
CK: Creatine kinase
Cre: Creatinine
GCS: Glasgow Coma Score
HDF: Hemodiafiltration
Mb: Myoglobin
PEPA: Polyester polymer alloy
RRT: Renal replacement therapy
SpO_2_: Peripheral capillary oxygen saturation
